# Estimating CO_2_ Emission Savings from Ultrahigh Performance Concrete: A System Dynamics Approach

**DOI:** 10.3390/ma14040995

**Published:** 2021-02-20

**Authors:** Mubashar Sheheryar, Rashid Rehan, Moncef L. Nehdi

**Affiliations:** 1 National Institute of Urban Infrastructure Planning, University of Engineering and Technology, Peshawar 25000, Pakistan; 14pwciv4066@uetpeshawar.edu.pk (M.S.); rashid@uetpeshawar.edu.pk (R.R.); 2 Department of Civil and Environmental Engineering, University of Western Ontario, London, ON N6A 5B9, Canada

**Keywords:** ultrahigh-performance concrete, ordinary Portland cement, concrete, system dynamics, modeling, carbon dioxide, greenhouse gas, sustainability, policy testing

## Abstract

Ordinary Portland cement concrete (OPC) is the world’s most consumed commodity after water. However, the production of cement is a major contributor to global anthropogenic CO_2_ emissions. In recent years, ultrahigh performance concrete (UHPC) has emerged as a strong contender to replace OPC in diverse applications. UHPC has much higher mechanical strength, and thus less material is used in a structural member to resist the same load. Moreover, it has a much longer service life, reducing the long-term need for repair and replacement of aging civil infrastructure. Thus, UHPC can enhance the sustainability of cement and concrete. However, there is currently no robust tool to estimate the sustainability benefits of UHPC. This task is challenging considering that such benefits can only be captured over the long-term since variables, such as population growth and cement demand per capita, become more uncertain. In addition, the problem of CO_2_ emissions from cement and concrete is a complex system affected by time-dependent feedback. The System Dynamics (SD) method has specifically been developed for modeling such complex systems. Accordingly, a SD model was developed in this study to test various pertinent policy scenarios. It is shown that UHPC can reduce cumulative CO_2_ emissions of cement and concrete—over the studied simulation period—by more than 17%. If supplementary cementitious materials are further deployed in UHPC and new technologies permit reducing the carbon footprint per unit mass of cement, emission savings can become more substantial. The model offers a flexible framework where the user controls various inputs and can extend the model to account for new data, without the need for reconstruction of the entire model.

## 1. Introduction

**Figure 1:**
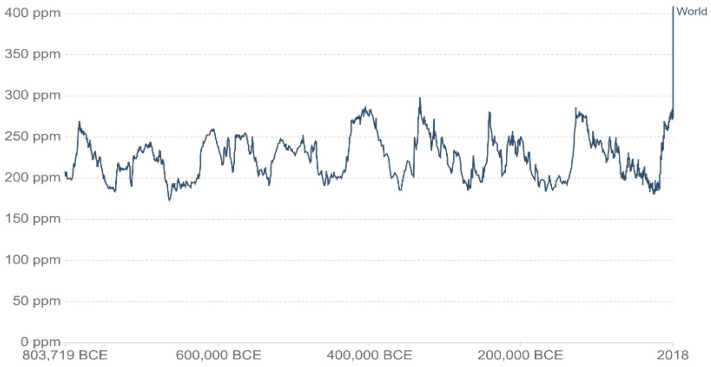
Long-term CO_2_ emissions measured in parts per million (PPM); adapted from [1].

The rise in the Earth’s surface temperature has become a great concern and a matter of considerable controversy [1]. It is largely believed to be caused by increased anthropogenic emissions of greenhouse gases (GHGs) such as methane and carbon dioxide. Solar radiations enter the Earth atmosphere as short waves. Radiations reflected back by Earth are typically longer wave and can become entrapped by GHGs, thus raising the global temperature and effecting climate change [2]. Among GHGs, CO_2_ constitutes about 20%, water vapor 60%, while the remainder portion consists of other minor gases like methane, N_2_O, etc. Human activities are generally responsible for about 63% of the total CO_2_ emissions [3]. Luthi et al. [4] conducted various experiments on ice cores and tried to estimate the CO_2_ concentration in the atmosphere over the last 800,000 years. Up to the last century, the concentration of CO_2_ in the atmosphere was less than 300 ppm [5] but increased significantly thereafter (Figure 1). With the industrial revolution, population growth, increased urbanization, and economic activity, there is greater demand to build new infrastructure and repair the existing one [4]. Various minerals and other materials are extracted from the earth crust and processed to meet the demands of urbanization and infrastructure development [6]. Concrete is the world’s most used commodity after water and by far the most used construction material [7]. Accordingly, about 8–10% of global CO_2_ emissions are contributed by Portland cement production [8] since producing one ton of cement clinker releases about 0.816 ton of CO_2_ in the atmosphere [9]. The world’s cement production in 2019 reached about 4.1 gigatons (Gt) and is expected to increase to 5.3 Gt in the year 2050 [10]. In 2019, China produced about half of the world’s cement (2.2 Gt), followed by India (0.320 Gt) [11].

To mitigate climatic change implications, researchers and policy makers have developed various strategies and approaches to reduce GHG emissions. For instance, the International Energy Agency (IEA) presented a number of methods to reduce carbon emissions up to the year 2060 [12]. These involve partial replacement of cement with supplementary cementitious materials (SCMs) [13], CO_2_ capture and storage [14], life cycle assessment methodologies [15], etc. A system dynamics tool was created by taking into account the replacement of Portland cement with SCMs like fly ash and blast furnace slag to reduce CO_2_ emissions [2]. More recently, ultra-high-performance concrete has emerged as a strong contender with much higher mechanical strength (around 150 MPa) [16] and a greater service life of about 150 years [17]. Depending on the admixtures used, steel fibers dosage, and curing methods applied, the cement content in ultrahigh performance concrete (UHPC) mixtures can vary significantly, for example, a range of cement content of 27–40% was reported in [18]. Similarly, the cement content in four commercial UHPCs (Ductal®, Chicago, IL, USA; Cemtec®, Enns, Austria; DURA®, Chemor, Perak, Malaysia; BSI®, Vélizy-Villacoublay, France) reported in [19] varied from 28.5% to 39.2%. In this study, a 22.7% dosage of cement was assumed for UHPC based on typical compositions of UHPC reported in [16]. These cement contents are significantly greater than that in conventional concrete. Owing to its higher compressive strength normally exceeding 150 MPa, the cross-section of UHPC structural members is substantially decreased, for resisting the same mechanical load, compared to that of counterparts made of ordinary Portland cement (OPC) with compressive strength usually ranging from 30 to 70 MPa [20]. The ultrahigh strength permits the design of slender structural members, leading to a reduction in the self-weight of structures due to less use of materials. Moreover, for the same reinforcement ratio, the amount of reinforcing steel will be decreased in smaller cross-section members, producing a further reduction in structural dead loads. In this study, it is assumed that owing to its superior mechanical strength, the volume of structural members made of UHPC can be reduced by about 50% compared to that of normal strength concrete counterparts [16]. Although the user of the model can modify this assumption through the user input interface for testing different policy scenarios, the 50% assumption is supported by existing practice. For instance, Hajek and Fiala [20] calculated the self-weight of floor slab alternatives made with OPC and UHPC. The self-weight of the 200 mm thick OPC slab was 491 kg/m^2^, while the equivalent UHPC slab design yielded a self-weight of 223 kg/m^2^, with a reduction in self-weight of 54.6%, which is very close to the 50% assumed in the present study. Moreover, Bernier et al. [21] tested full-scale structural elements made of ultrahigh-strength fiber-reinforced concrete, including columns, beams, and beam-column joints. They concluded that UHPC members with a cross-section of 50, 000 mm^2^ can be compared to members with a cross-section of 210,000 mm^2^ made of conventional reinforced concrete. This represents savings of up to 76% in materials, which is beyond the 50% assumed in the present study. Therefore, there is a need for a rational tool that can estimate the effect of using UHPC for infrastructure construction on the CO_2_ emission reductions resulting from such material savings, enhanced durability, and less demolition and replacement of structures.

UHPC is a brittle material. However, when 2.5–3% of steel microfiber is added to the cementitious matrix, greater enhancement in ductility can be achieved. Moreover, the much denser microstructure of UHPC hinders the ingress of chloride ions and other hostile substances into the cementitious matrix, greatly improving the durability of UHPC structures. UHPC mixtures mostly consist of fine materials with denser particle packing, thus eliminating the weak coarse aggregate cement matrix transition zone, which further enhances strength and durability [22]. The initial cost of UHPC is greater than that of conventional concrete. A feasibility study commissioned by the Nevada Department of Transportation [23] reported that the cost of UHPC, on a volumetric basis, in North America was more than 11 times higher than that of OPC. However, the report also noted that this gap in cost is expected to shrink with growing adoption of UHPC in the continent while noting that in Europe, UHPC is about 4 times costlier than OPC. When long-term maintenance and repair costs are also taken into consideration in addition to the initial costs, UHPC can be considered more cost effective based on a life cycle analysis [17].

Accordingly, a rational understanding of the implications of using UHPC on CO_2_ emissions from cement production has paramount importance and would be helpful for policy making regarding the mitigation of climate change. However, the demand for cement, the durability of structures, CO_2_ capture in concrete via carbonation, and other pertinent aspects make this a complex system characterized by time-dependent feedback. Thus, traditional predictive tools and modeling approaches will not be efficient. The system dynamics (SD) technique has been adopted by researchers in various fields including global warming [24], ecological and economic sustainability [25], planning and management [26], etc. The SD approach can be used for analyzing CO_2_ emissions from cement under different circumstances or policy scenario through interactive feedback loops.

In this paper, SD modeling is used for capturing the environmental impact of the substitution of UHPC for ordinary Portland cement concrete (OPC). A survey of the open literature indicates that there is presently no such quantitative tool for modelling GHG emissions resulting from the replacement of OPC with UHPC. Thus, this proposed SD tool could help policy makers to estimate the GHG emission reductions under various economy growth and policy intervention scenarios. This approach should garner greater interest in SD modeling of GHG emissions and blaze the trail for policy makers such as the World Business Council for Sustainable Development (WBCSD), the Cement Sustainability Initiative (CSI), and the Intergovernmental Panel on Climate Change (IPCC) to explore the mitigation of CO_2_ emissions by involving UHPC incorporating SCMs in policy making.

## 2. Modeling Strategy and Model Building

### 2.1. System Dynamics Approach

Decisions are often taken by policy makers to stabilize a system; yet the system often destabilizes itself after some time. Forrester [27] named this mechanism a “counterintuitive system”. He developed a paradigm called ‘system dynamics’ to solve such problems that involve time-dependent feedback or counterintuitive behavior [27]. Real world issues usually exhibit unintended behavior; yet they are traditionally solved without incorporating feedback generated from the problem, which leads to unanticipated consequences. The SD approach can be helpful in modelling systems which involve unanticipated consequences or non-linearity [25]. Qualitative and quantitative approaches are used in SD to study complex systems. The qualitative approach is based on systems thinking, while the quantitative approach uses stock and flow diagrams to represent and solve the system numerically.

### 2.2. Causal Loop Diagram

A causal loop diagram (CLD), or influence diagram, qualitatively illustrates causal relationships among various variables of a system. A causal relationship of one variable on another is represented with an arrow symbol. A positive sign (+) placed at the arrowhead in the diagram shows a direct relationship between the variables, i.e., an increase or decrease in one variable causes an increase or decrease, respectively, in the other variable. Similarly, a negative sign (−) shows a relationship that is characterized by a causal effect in the opposite direction, i.e., an increase in one variable causes the other variable to decrease and vice versa. Construction of CLDs often reveals the existence of feedback loops in dynamic systems. A feedback loop exists when causal relationships exist such that an initial perturbation in one variable ultimately causes a further change in the initiating variable. Feedback loops can be positive (reinforcing) or negative (balancing).

#### 2.2.1. Reinforcing Loop or Positive Feedback

**Figure 2:**
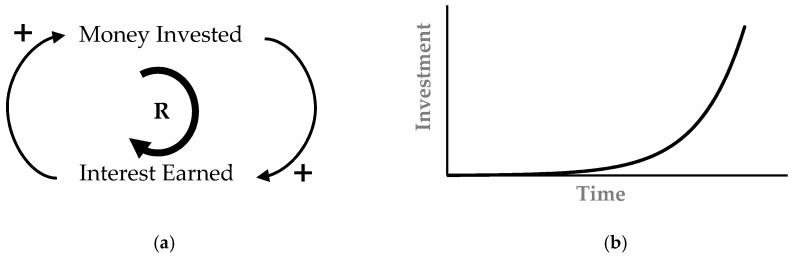
Reinforcing feedback loop: (**a**) causal loop for reinforcing loop, and (**b**) exponential growth due to reinforcing loop.

A reinforcing loop depicts an exponential growth behavior, where increasing the quantity of one variable causes the increase of the other variable, which again effects the variable in the same direction. An example illustrating positive feedback is academic research. If a student shows good performance in research, this increases the supervisor support, which further enhances the student’s research capability. Another classical example is compounding interest, as shown in Figure 2a; the greater the amount of money invested, the greater will be the interest earned, that in turn gets added to the money invested, thereby yielding even still greater interest. Sometimes positive feedback also yields self-reinforcing declining behavior, in contrast to exponential growth. For example, the loss in business decreases the confidence level, which again affects loss in the same direction.

#### 2.2.2. Balancing Loop or Negative Feedback

**Figure 3:**
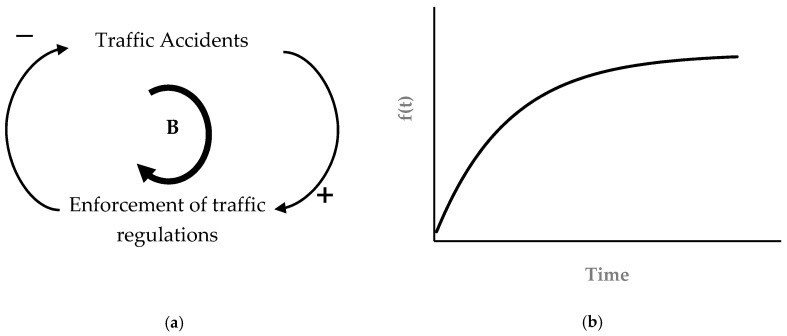
Balancing feedback loop: (**a**) causal loop for a balancing loop, and (**b**) goal seeking behavior due to balancing loop.

Negative feedback moves the system towards equilibrium. As an example, consider the situation where the number of accidents on a certain highway increases, the authorities respond to this rise through stricter enforcement of traffic laws, which helps lower the number of accidents. Thus, the initial rise in accidents ultimately causes their decline as the feedback effect traverses through the system (Figure 3a). Negative feedback processes seek balance and equilibrium and bring stability to the system. Any initial disturbance that tends to move the system away from equilibrium is counteracted by the negative feedback that strives to attain a predetermined goal.

Usually, most systems comprise more than a single feedback loop and the overall system behavior arises as a consequence of the various interacting feedback loops. Thus, in addition to the exponential growth and goal seeking behaviors illustrated above, systems can exhibit other behaviors such as oscillation, S-shaped growth, overshoot, and collapse, etc.

### 2.3. Causal Loop Diagram for UHPC Adoption

**Figure 4:**
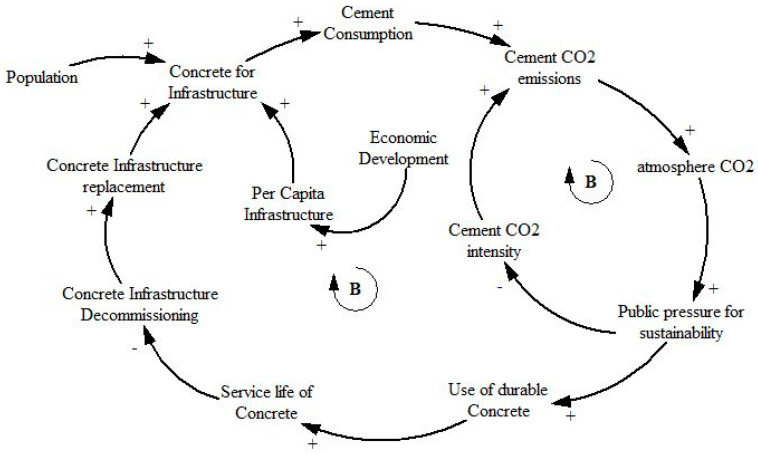
Causal loop diagram for ultrahigh performance concrete (UHPC) adoption process.

The benefits of UHPC are well known, and the adoption of UHPC in place of OPC could contribute to sustainable development through reducing CO_2_ emissions owing to enhanced life cycle performance of concrete structures. However, the transition from OPC to UHPC may not be an easy and quick process. The system comprising the use of OPC and UHPC involves diverse and interconnected variables. A change in one variable can have cascading effects on the other variables involved in the system. Thus, it is possible that policy implementation may be thwarted or even defeated if all the variables and their interrelationships are not properly accounted for. It is therefore imperative to construct and study a UHPC adoption system in a holistic manner. Such a model has a value on its own that it can help identify potential feedback loops in the system. A simplified CLD for UHPC adoption is thus developed and presented in Figure 4. It is a simplified CLD in that the direct causal relationship between two variables shown herein may in fact have additional intermediate variables, which for the sake of brevity and avoiding clutter, have been omitted. As long as the omission does not introduce an error in the polarity of a causal relationship, the simplified connection is deemed justified. For example, if variable A affects variable B through several intermediate variables and the direction of polarity (+ or − signs placed at the arrow heads) through the entire causation chain is such that an increase in variable A ultimately effects an increase in variable B, then it is not incorrect to represent the whole chain in a condensed form as simply a causal relationship between A and B, the arrow head with a + sign terminating at variable B.

Figure 4 shows that concrete required for infrastructure development is influenced by two main variables: Population growth and per capita infrastructure requirement. As population grows, the need for providing shelter and other physical infrastructure grows. Similarly, the more a community becomes economically developed, the greater its demand for infrastructure on a per capita basis would be, which in turn leads to increased infrastructure. Portland cement is an essential ingredient of concrete since it is the binder and has significant importance because of its carbon and energy intensity. Thus, creation of new concrete infrastructure causes cement consumption to increase. The manufacture of cement is an energy intensive process that is also associated with colossal CO_2_ emissions. The process of cement manufacture from its constituent raw materials causes the release of CO_2_ gas into the atmosphere. Total CO_2_ emissions due to cement manufacture are a function of the total quantity of cement produced and the carbon intensity of cement on a per unit weight basis. This carbon intensity can be reduced through various approaches, a few of which involve employing less carbon intensive fuels and adopting more energy efficient processes, as well as replacing part of the Portland cement with other SCMs. Thus, the carbon intensity of cement is not a static entity and can evolve over time. For example, the amount of CO_2_ emitted per kg of cement produced has declined from about 1 kg two decades ago to about 0.82 kg today. It is reasonable to assume that the higher the concentration of CO_2_ in the atmosphere, the greater would be the public pressure (reflected through government policies) to reduce the carbon intensity of cement. A balancing feedback loop is thus formed between cement CO_2_ emissions, atmospheric CO_2_ concentration, public pressure for sustainability, cement CO_2_ intensity, and cement CO_2_ emissions.

Another consequence of public pressure towards sustainability is manifested in terms of adoption of durable concretes, such as UHPC. Durable concrete has a longer service life and hence reduces the need for repair and earlier decommissioning (and thereby reconstruction) of infrastructure. This ultimately leads to a reduction in the creation of infrastructure from concrete, closing the loop that is balancing in nature.

### 2.4. UHPC Carbon Emissions Model

**Figure 5:**
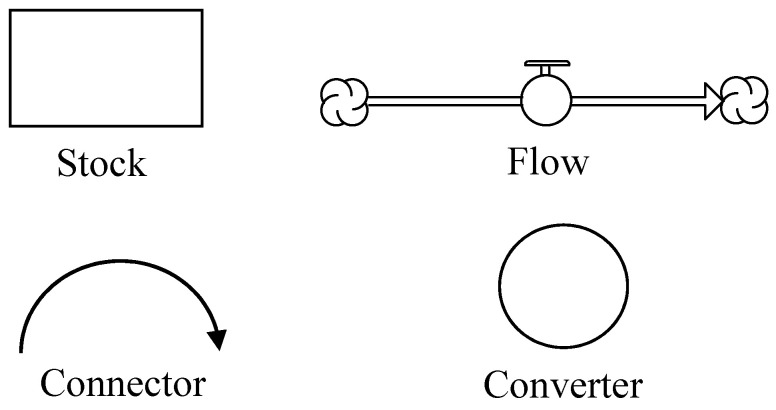
Building blocks of system dynamics models in Stella.

To implement the qualitative causal loop diagram of the UHPC carbon emissions system shown in Figure 4, a mathematical model was developed in the Stella environment (isee systems, 9.1.4, Lebanon, NH, USA) [27]. The basic building blocks of a system dynamics model include (Figure 5): Stocks; Flows; Convertors; and Connectors.

Stocks represent accumulations—both physical and non-physical. Examples of physical stocks are the amount of water in a reservoir, the total amount (by volume or weight) of concrete infrastructure in a region, the total concentration of CO_2_ gas in the atmosphere, etc. A non-physical stock represents the aggregate political pressure of a community to reduce GHG emissions. Stocks represent the ‘traces’ left by an activity. Material in a stock exists at a given point in time and persists even when activities cease. Flows represent activities or actions in a stock that transport quantities into or out of a stock instantaneously or over time. Examples of flows are the annual production of cement, decommissioning of concrete structures upon completing their service life, and monthly revenues or expenditures of a construction firm. Converters are used in SD models as objects to house mathematical equations or functions. Conversely, connectors are used to convey information among the three elements described earlier. For easy comprehension of the model structure, the developed model is organized into four main sectors. Elements within each of these sectors are connected to elements in other sectors, and hence, the sectors are merely artificial constructs used for ease of description. Each of these sectors is first shown individually for better legibility, and then later presented together as one integrated model. The model sectors are discussed in the following sub-sections:

#### 2.4.1. Cement Demand Sector

**Figure 6:**
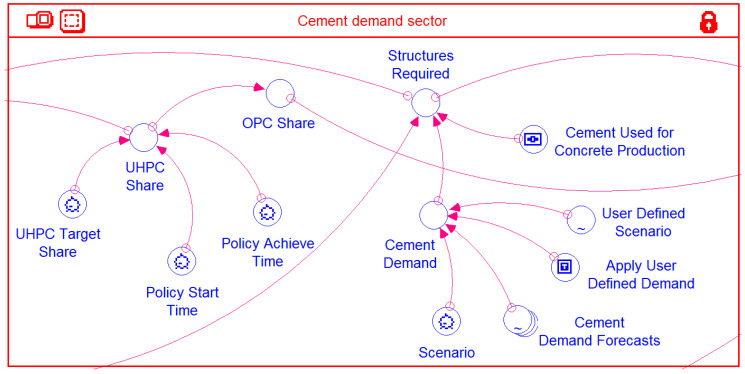
Cement demand sector of the model.

This sector (Figure 6) only contains converters, which are linked amongst themselves as well as with elements of the other sectors via connectors and essentially provides inputs to the model. Connectors/arrow lines emanating from the converters *Structures Required* and *OPC Share* and the one terminating at the former appear cutoff at the sector outline. However, these lines in fact continue and are connected to model elements in other sectors. This is also the case for other connectors in the other sectors, which apparently terminate at the sector outline, but in fact continue and connect with elements in the other sectors. Figure presented later in this text depicts how all these interconnections extend across the various sectors.

**Figure 7:**
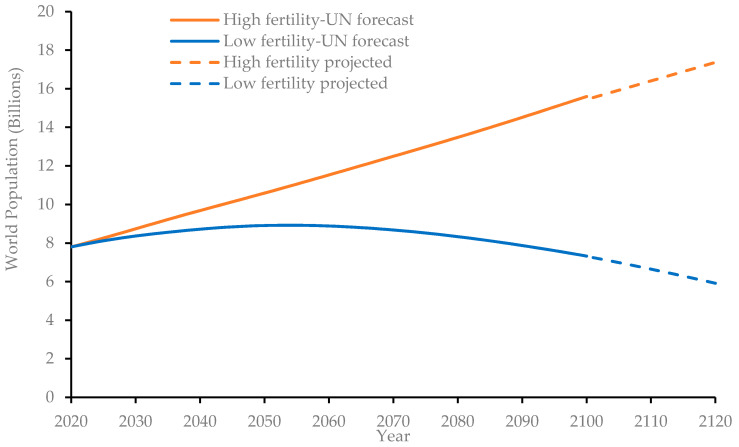
World population forecasts.

The main input comprises cement demand forecasts over the simulation horizon, i.e., years 2020 to 2120. Following the methodology adopted by Chaturvedi and Ochsendorf [28], future cement consumption trends are modelled as a function of the world population. The United Nations (UN) considered low, medium, and high fertility rates to project world population for the next eight decades, i.e., until 2100 [29]. Amongst these three main scenarios, the low and high fertility rates were selected in the present study for enveloping the possible population growth scenarios. Since the selected simulation period for this study is 100 years, the United Nations population forecasts needed to be projected further by another 20 years, which was accomplished using regression analysis. The equations derived through regression analysis have high R^2^ values—0.9994 and 0.9993 for the high- and low-fertility population growth trends, respectively. Figure 7 shows that the global human population is expected to grow to 15.6 billion by the turn of the century under the high-fertility growth assumption. For the low-fertility assumption, the world population is estimated to peak at 8.92 billion in the year 2054, then start to decline to about 7.32 billion by the year 2100. Extrapolating these trends further, it was estimated that the population figures under these two scenarios will reach 17.4 billion (high-fertility) and 5.9 billion (low-fertility).

**Figure 8:**
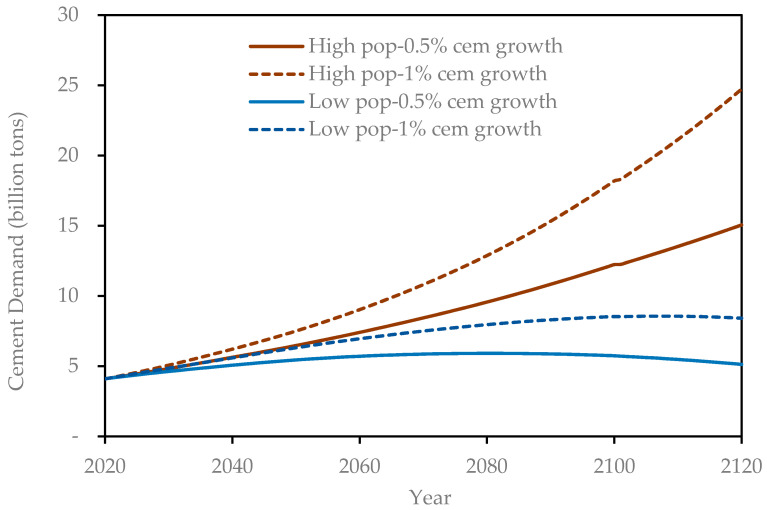
Global cement demand scenarios.

Developed countries have recently exhibited low growth rates in the per-capita cement consumption, while those developing nations that have lagged in the provision of infrastructure to their populations have rather shown high growth rates. Accordingly, in their estimates for future world demand of cement, Chaturvedi and Ochsendorf [28] used growth rates of 5% and 10% in per-capita cement consumption in conjunction with the UN population forecasts. Since in the present study a much longer simulation horizon is adopted, it is reasoned that growth rates of 5–10% cannot realistically be sustained over such a long period. Accordingly, two growth scenarios for per capita cement consumption are assumed, 0.5% and 1% per year, each of which is assumed to remain constant over the 100-year simulation period. Each of these growth scenarios were used in conjunction with both population growth scenarios (Figure 7) to estimate the global cement consumption over the simulation horizon. The resulting four scenarios are shown in Figure 8.

The four cement demand curves shown in Figure 8 are used as built-in scenarios in the Stella model through the converter *Cement Demand Forecasts*. A model user will have the option to select any one of these default curves or enter their own separate cement demand curve through the converter *User Defined Scenario*. Though most of the cement is used in concrete production, there are other minor uses of cement, such as soil stabilization, mortar production, etc. Accordingly, it is assumed that 90% of the cement consumed will be used for concrete production. This default value can be changed by the user to any other value through the model object *Cement Used for Concrete Production*. The variable *Structures Required* (in Figure 6) is used to calculate the amount (in tons) of concrete produced, based on the corresponding value of cement consumption for any simulation year and the choice of cement consumption curve (Figure 8) adopted. OPC contains about 13.5% of cement by weight and accordingly, the production of OPC is calculated. Another simplifying assumption is that the concrete produced is either OPC or UHPC. In the absence of any deliberate policy choice, all the *Structures Required* would be made of OPC and thus *OPC Share* would be 100%, while that of UHPC would be 0%. The user has to generate a policy scenario whereby the *UHPC Share* will increase to a specified fraction (*OPC Share* decreasing correspondingly) beginning in a given simulation year and the target *UHPC Share* achieved over an implementation period specified through *Policy Achieve Time*.

**Figure 9:**
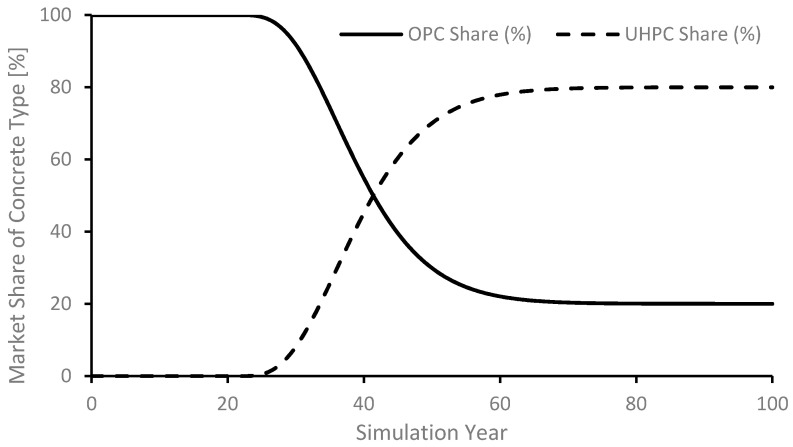
Transition implementation of policy target.

It is assumed that real world policies cannot be implemented overnight and are rather characterized by an initial slow up take, followed by a period of rapid adoption, then exhibit a slowing down as the target nears. In other words, even successful policies are characterized by an S-shaped trend. This is implemented in the model through an exponential smoothing function (built-in function in Stella) where the target *UHPC Share* is achieved as an S-shaped trajectory. Figure 9 shows an example wherein the *UHPC Share* is set to reach a value of 80% from an initial value of 0%, beginning in year 20 and achieved over a 20-year period. It may be noted that the sum of the OPC and UHPC shares remains 100% throughout the simulated period, a consequence of the simplifying assumption, mentioned above, that only these two types of concretes are made for meeting the requirements of infrastructure.

#### 2.4.2. OPC Sector

**Figure 10:**
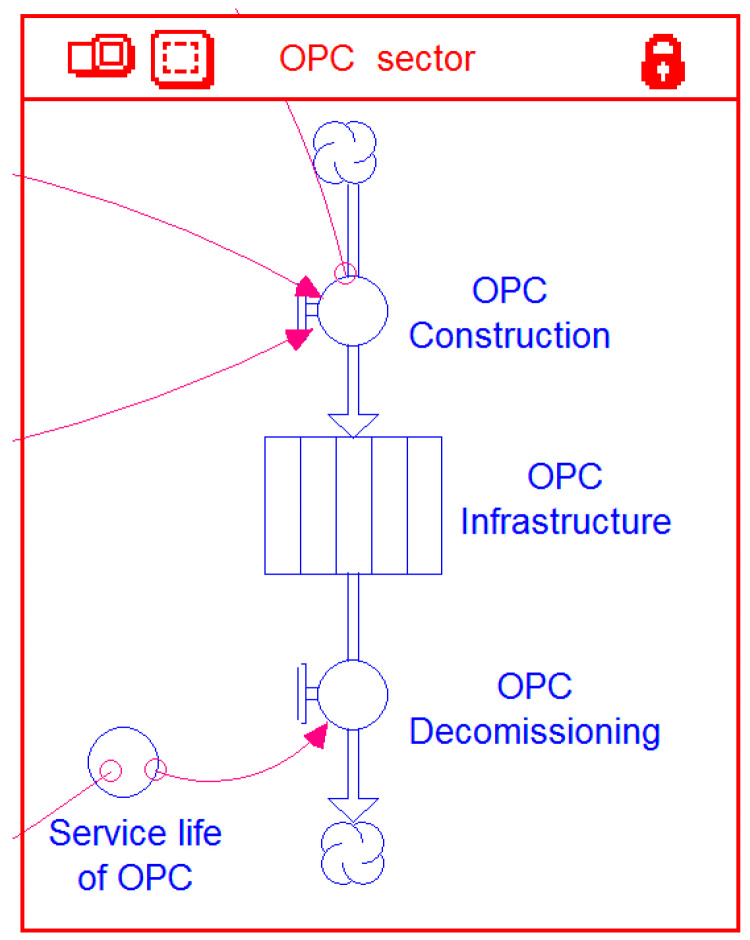
OPC (ordinary Portland cement) sector of the model.

To keep track of how much OPC is manufactured annually during the simulation period, a dedicated sector named *OPC Sector* is included in the Stella model (Figure 10). The sector includes a single stock-flow structure. The stock *OPC Infrastructure* represents the amount of infrastructure (in metric tons) that is made of OPC. The initial value— at the start of the simulation—of this stock is set to zero. Once the simulation starts, the inflow *OPC Construction* begins adding the annual production of OPC to the stock. A built-in feature of the Stella software allows the constructed OPC to stay within the stock for a period specified as the *Service life of OPC* and then gets removed through the *OPC Decommissioning* outflow.

#### 2.4.3. UHPC Sector

**Figure 11:**
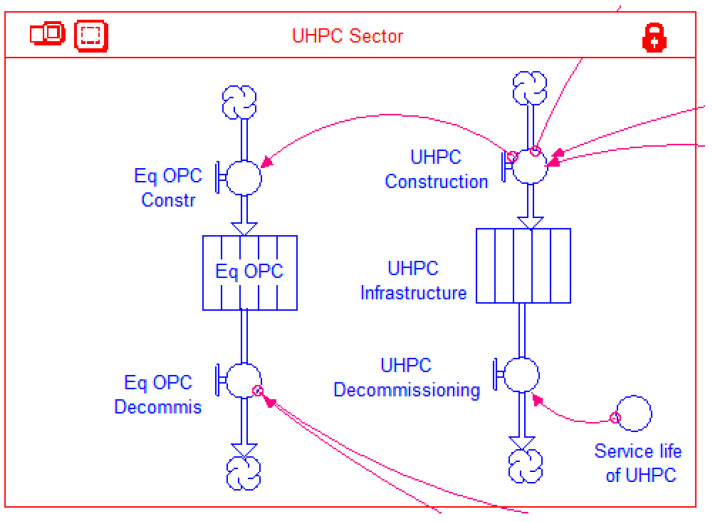
UHPC sector of the model.

The *UHPC Sector* shown in Figure 11 has essentially the same stock-flow structure as that for the *OPC Sector*, but with an additional stock-flow structure. This latter structure is comprised of an inflow *Eq OPC Constr*, a stock *Eq OPC*, and an outflow *Eq OPC Decommis*. These model elements do not have a physical significance, but are included to capture the effect of longer service life of UHPC. It is assumed that each of the global cement demand projections (Figure 8), besides catering to newly constructed infrastructure, also includes a fraction to replace the aging concrete infrastructure that reaches the end of its service life and is decommissioned. Whence a shift is made to the more durable UHPC, the decommissioning of infrastructure made of UPHC would be delayed as compared to that of OPC. The default service life of UHPC has been assumed as 150 years in this study as per [17], while the service life of OPC has been assumed as 50 years. Thus if *x* tons of infrastructure are constructed with UHPC in year *t*, concrete that would have been required to replace *2x* tons of OPC in year *t* + 50 would no longer be needed. As stated earlier, because of its higher compressive strength, the amount of UHPC needed to construct the same infrastructure is half the amount of OPC. Thus, when *x* tons of UHPC are constructed, the equivalent OPC that it has replaced is *2x* tons. Finally, the cement demand would also be reduced proportionately in the year *t* + 50. It should be noted that infrastructure components, such as buildings and bridges, do not get necessarily demolished and replaced precisely at the end of their service life. In most cases, such infrastructure remains in use after applying necessary rehabilitation and retrofitting techniques, sometimes accompanied with load/use restrictions. However, the complexity of accounting for the diverse kinds of rehabilitation/retrofitting techniques, the extent of their application (i.e., how widespread each of these are), and their respective effects on prolonging the continuing use of the infrastructure, is beyond the scope of the present study. Hence, it is assumed that the infrastructure on reaching the end of its design service life gets demolished and replaced with new infrastructure. Though a simplifying assumption, it may be noted that it is applied to both the OPC and UHPC infrastructure, hence, as far as the objective of the study is to make a comparative assessment of CO_2_ emissions for the two types of concretes, the results obtained should still offer useful insights.

#### 2.4.4. CO_2_ Emissions Sector

**Figure 12:**
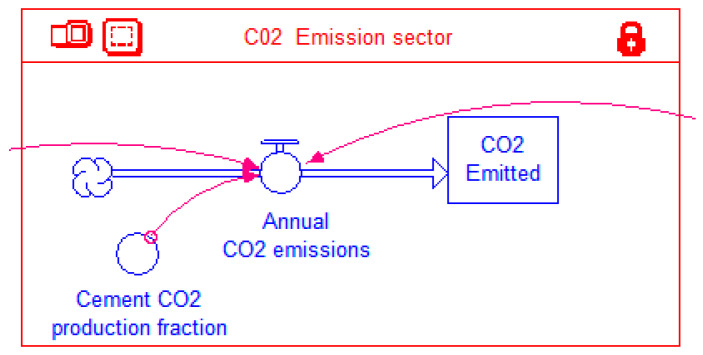
CO_2_ emissions sector of the model.

Information from the UHPC and OPC sectors is transmitted to the *CO_2_ Emissioins Sector* that is shown in Figure 12. This sector has a stock *CO2 Emitted* that represents the cumulative CO_2_ emissions over a simulation period. The annual contributions to this stock from the cement industry are added through the inflow *Annual CO2 emissions*. These annual emissions are in turn contributed by the cement used in manufacturing OPC and UHPC. It is assumed that each ton of UHPC and OPC consumes 22.7% and 13.5% of cement by weight, respectively. The total amount (weight) of cement consumed during a year is multiplied by the factor *Cement CO2 production fraction* that represents the amount of CO_2_ released per unit weight of cement consumed. A value of 0.85 kg of CO_2_ per kg of cement (approximately taken an average of 0.816 tons [9] and 0.900 [30] tons per ton of clinker) is assigned by default to this factor in the model. However, this can be changed by the user at the model interface level. Thus, for each ton of OPC and UHPC infrastructure, the CO_2_ burden is 0.11 and 0.19 ton, respectively. However, as mentioned above, to construct the same amount of infrastructure, the amount of UHPC required is half that of the OPC. Hence, on a per unit of infrastructure basis, CO_2_ emissions are reduced by 16% when UHPC is used instead of OPC.

Atmospheric CO_2_ eventually dissolves into the oceans over a period of 20–200 years [31], and hence theoretically the stock of CO_2_ emitted to the atmosphere by the cement industry should deplete over time. However, given the large range of the time period involved, the inherent uncertainty and continuing annual additions to the stock, a simplifying assumption in this study is that the stock of atmospheric CO_2_ emissions does not deplete. Accordingly, no outflow is provided for the *CO2 Emitted* stock (Figure 12).

**Figure 13:**
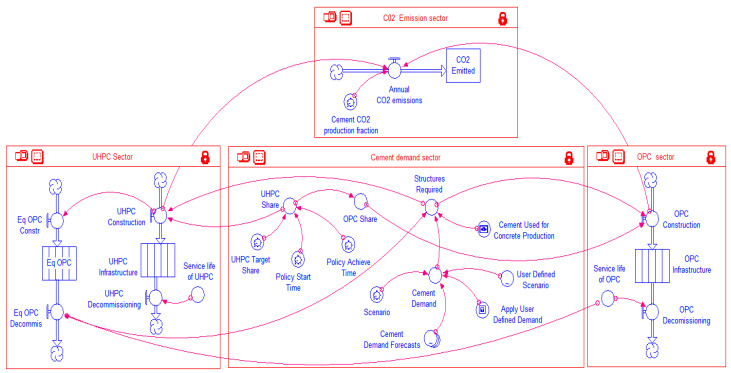
Full Stella model.

The complete model comprising the four sectors described above is shown in Figure 13. While the basic building blocks of the model can hardly be legible in this complex figure, they have already been described in the individual sectors in figures above. The purpose of this figure is rather to illustrate the overall interconnectedness of the four sectors.

## 3. Results

### 3.1. Simulation Scenarios

To demonstrate the utility of the model in terms of how a shift towards increased adoption of UHPC can help achieve savings in CO_2_ emissions and thereby contribute towards sustainable development, several scenarios are created. First, four main scenarios are assumed based on the projected cement consumption trends shown in Figure 8 above. These are summarized in Table 1 below:

Further sub-scenarios are then created for each of these four main families with the following features:

1. A base case scenario with only OPC being used to meet the infrastructure demand, i.e., the share of OPC remains 100% throughout the simulation period (sub-scenario I).
2. Simulation starts with 100% share of OPC and the target for UHPC share is set at 50%. The implementation of this policy target starts in year 0 and is planned to be achieved over a 25-year horizon (sub-scenario II).
3. Simulation starts with 100% share of OPC, but starting in year 0, it is planned to be completely replaced by UHPC over a 50-year period (sub-scenario III).
4. Simulation starts with 100% share of OPC, but starting in year 0, it is planned to be completely replaced by UHPC over a 25-year period (sub-scenario IV).

**Figure 14:**
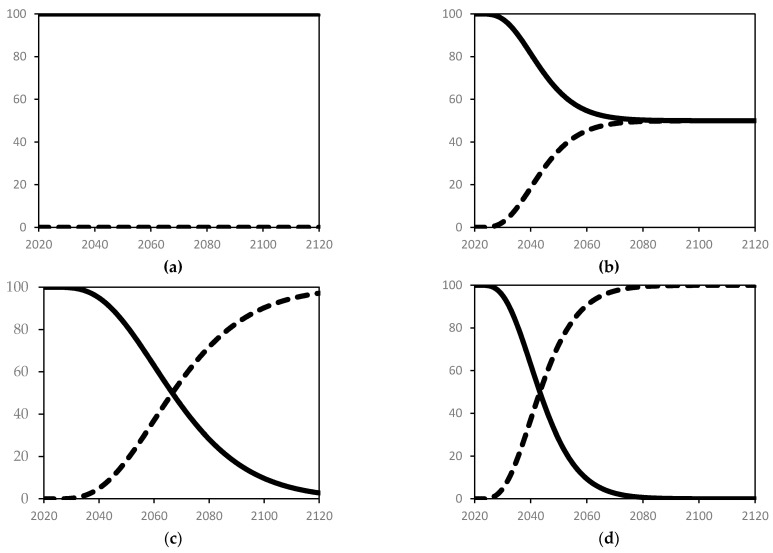
Share of concrete type for infrastructure. In all four panels: Solid and dashed lines represent OPC and UHPC shares, respectively; the vertical axis is the fraction of concrete type (%); and the horizontal axis is the simulation time [years]. (**a**) Sub-scenarios I; (**b**) Sub-scenarios II; (**c**) Sub-scenarios III; and (**d**) Sub-scenarios IV.

These four sub-scenarios illustrated in Figure 14 are intended for policy testing and are not predictions of actual future trends. Sub-scenarios III and IV—with 100% targeted share for UPHC—may not be realistic to achieve due to technological and economic constraints. Yet, these are included as illustrative scenarios to explore the full potential of realizing reductions in CO_2_ emissions. With the full spectrum of possible reductions at hand, policy makers can then choose to set any intermediate targets, or even devise policies that help overcome the economic barriers and/or may choose to invest in research to reduce, if not eliminate, such technological barriers. Each panel in Figure 14 shows the share of OPC and UHPC as part of the total infrastructure. Thus, the model was run for a total of 16 cases (4 main scenarios × 4 sub-scenarios). The results are presented in the following section.

### 3.2. Simulation Results

#### 3.2.1. Annual CO_2_ Emissions

**Figure 15:**
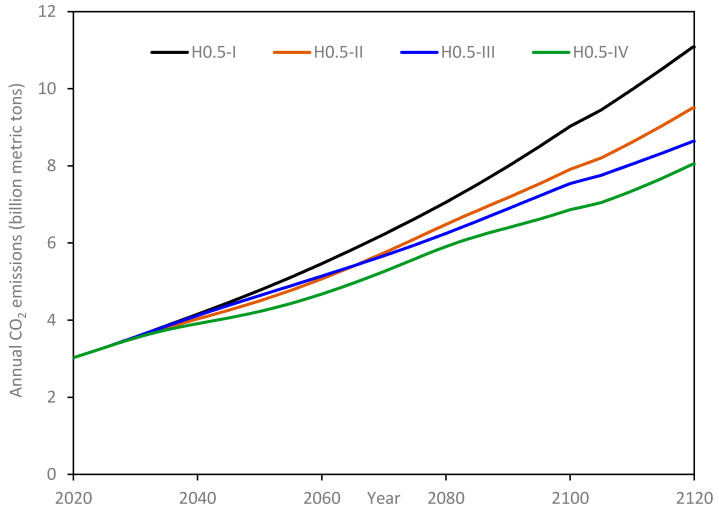
Annual CO_2_ emissions for H-0.5 scenarios.

**Figure 16:**
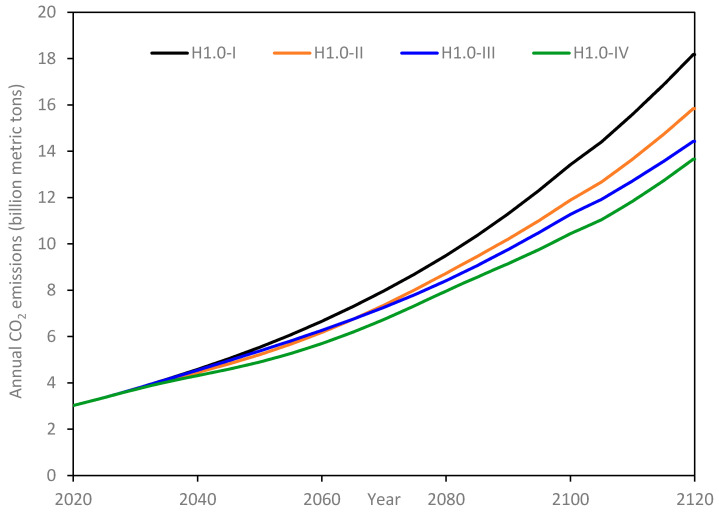
Annual CO_2_ emissions for H-1.0 scenarios.

**Figure 17:**
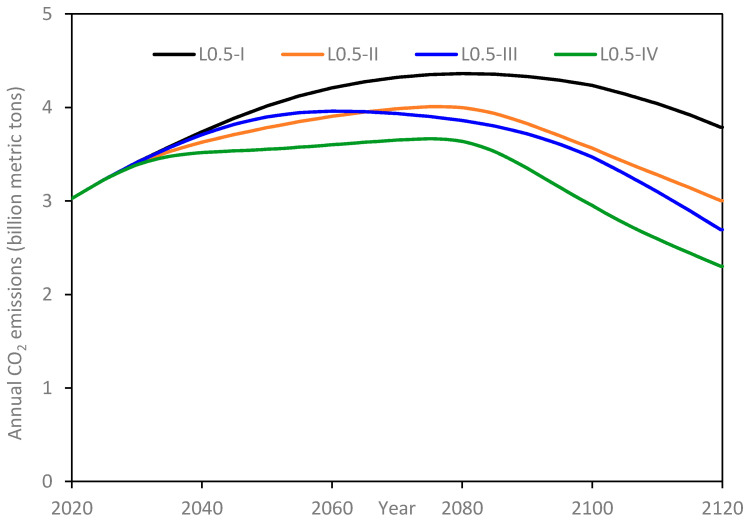
Annual CO_2_ emissions for L-0.5 scenarios.

**Figure 18:**
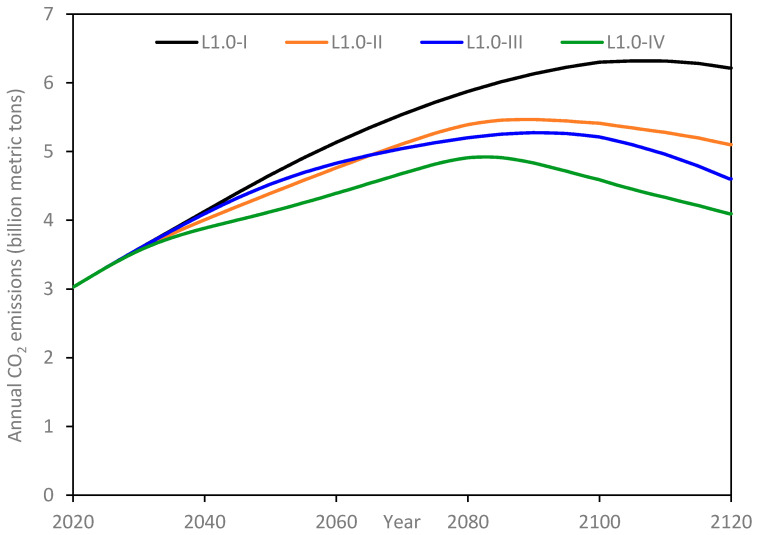
Annual CO_2_ emissions for L-1.0 scenarios.

Simulation results for the four sets of scenarios are presented in Figure 15, Figure 16, Figure 17 and Figure 18. It should be noted that these results are plotted with varying scales along the y-axis so that trends followed in individual sub-scenarios are clearly discernable. The annual CO_2_ emissions for H-0.5 scenarios are depicted in Figure 15. Scenario H0.5-I is shown as a reference wherein the infrastructure is assumed to be constructed using OPC alone. In this scenario, the global CO_2_ emissions from the concrete industry start at about 3 billion metric tons and just exceed 11 billion metric tons by the end of the simulation period. This more than 3-fold increase in emissions results from the combined effect of population growth (2.23 times increase) and growth in per-capita demand for infrastructure compounded over the entire simulation period (1.005^100^ = 1.65). Sub-scenario II is generated by assuming that half of OPC is replaced with UHPC, the replacement occurring during the first quarter of the simulation. Accordingly, by the end of the simulation, the annual CO_2_ emissions reach 9.5 billion metric tons, i.e., a reduction of 14% when compared to the final year emissions of the H0.5-I scenario. Based only on the replacement of half of the OPC with UHPC and the associated reduction in cement consumption, the reduction in CO_2_ emissions should have been 8%; the additional 6% savings in CO_2_ emissions are realized due to the longer service life of UHPC, which decreases the infrastructure requirements to replace (end of service life) decommissioned infrastructure (see the discussion in Section 2.4.3). These savings are expected to grow further beyond the end of the simulation period.

In scenarios H0.5-III and H0.5-IV, all the OPC is targeted for replacement with UHPC, and the target slated to be achieved in the initial half, and initial quarter of the simulation period, respectively. Accordingly, in comparison with H0.5-I, the annual CO_2_ emissions, at the simulation end, are reduced by 22% and 27% under the H0.5-III and H0.5-IV scenarios, respectively. Although only half of OPC is targeted for replacement in Scenario H0.5-II, annual emissions under this scenario remain lower compared with that of Scenario H0.5-III having a complete OPC replacement target. This is a direct consequence of the longer policy implementation period chosen for the H0.5-III scenario. On the other hand, Scenario H0.5-IV has the same policy implementation time as that of H0.5-II and hence annual emissions for the former remain consistently below those of the latter.

Results for scenarios H-1.0 are shown in Figure 16. The general trend for all the sub-scenarios remains similar to those under H-0.5; the differences in absolute values are mainly driven by the higher growth rate in per capita consumption of 1% per annum assumed for H-1.0.

Figure 17 illustrates the results for the L-0.5 scenarios. With infrastructure demand assumed to be met only through OPC, Scenario L0.5-I exhibits a steady increase in annual emissions until year 2080, whilst achieving a peak value of 4.63 billion tons. In the meanwhile, population has started declining since the year 2054 (Section 2.4.1) due to the assumed low fertility scheme. However, the rate of population decline is offset by the growth in per capita infrastructure demand. It is only when the population decline accelerates sufficiently that the annual emissions also start a downward trend after the year 2080. Finally, by the end of the simulation period, annual emissions attain a value of 3.8 billion tons. Under Scenario L0.5-II, with 50% of OPC being replaced with UHPC and hence the concomitant decrease in cement consumption, with a peak value of about 4 million tons in annual emissions attained in year 2075. Thereafter, a sharply decreasing trend prevails, and by the end of the simulation period, annual emissions reach 3 billion tons, a similar value to that at the start of the simulation. Annual emissions under Scenario L0.5-III peak at 3.96 billion tons in year 2060 and finally end up 10% lower than the initial value at 2.7 billion tons. Annual emissions under Scenario L0.5-IV peak at 3.67 billion tons in year 2075, the same year during which L0.5-II scenario attained peak value. It may be recalled that both scenarios II and IV have the same policy implementation period of 25 years. By the end of the simulation, annual emissions have reduced by almost a fifth of the starting value (3 billion tons) to reach a final value of 2.3 billion tons.

Annual emissions for the L-1.0 scenarios are presented in Figure 18. Although these scenarios have the same underlying population growth projections, the growth rate in the per capita infrastructure at 1% per year is double that of the L-0.5 scenarios. Accordingly, the peaks in each sub-scenario are shifted towards the right, i.e., delayed. The trends under each sub-scenario in L-1.0 are qualitatively similar to the corresponding ones under the L-0.5 scenarios, albeit with higher values of annual emissions, as would be expected. Unlike the 3 sub-scenarios in L-0.5, none of the sub-scenarios in L-1.0 finish at or below the annual emissions at the start of the simulation.

In Table 2, it can be observed that for the L-0.5 scenario, with reference to its sub-scenario I, sub-scenarios II, III, and IV show the highest reductions in annual CO_2_ emissions, while the lowest reductions are manifested within the H-1.0 sub-scenarios. The trend among the four main scenarios, in order of increasing reductions for the corresponding sub-scenarios, is H-1.0, H-0.5, L-1.0, and L-0.5. Referring to Figure 8, it can be observed that this is the same order as that of main scenarios in terms of decreasing cement consumption. From this observation, one may conclude that although the adoption of UHPC instead of OPC shall result in a reduction in CO_2_ emissions, the proportionate impact diminishes as the overall infrastructure demand increases, whether driven by population growth, higher per capita demand for infrastructure, or both.

#### 3.2.2. Cumulative CO_2_ Emissions

Cumulative CO_2_ emissions over the simulation period are shown in Table 3. As would be expected, because of the estimated growth in cement consumption (Figure 8), the highest cumulative emissions occur under scenarios H-1.0, H-0.5, L-1.0, and L-0.5, in that order. Decreasing reduction in CO_2_ emissions occurring due to shifting to UHPC from the exclusive use of OPC (sub-scenario I) is observed for scenarios L-1.0, L-0.5, H-0.5, and H-1.0 (when scenarios II and IV are compared to scenario I). However, when scenario III is compared to scenario I for reductions in emissions, the order in terms of decreasing reductions is H-1.0, H-0.5, L-1.0, and L-0.5.

## 4. Discussion

Future CO_2_ emissions from the cement and concrete industry depend upon the growth in demand for infrastructure, which in turn is driven by population and economic growth. Accordingly, reasonably accurate estimates of both the future population growth and economic development are needed. There are inherent uncertainties in either of these variables. The uncertainty becomes magnified, and hence the problem more daunting when the projected time period for these estimates becomes longer. However, a longer simulation horizon is unavoidable for several reasons. First, the effect of GHG on climate change can only be reasonably discerned over the long term. Annual or even decadal analysis of GHG emissions is not very useful in climate change science. Accordingly, future climate change scenarios, such as the ones published by the IPCC, are often presented over horizons of about a century.

Furthermore, the demand for infrastructure (and hence cement/concrete) is not only for new construction, but also for reconstruction/replacement of outdated/demolished infrastructure. Thus, differences in service life of various concretes will have noticeable effects on the future requirements of concrete for building infrastructure. The service life of OPC and UHPC is roughly estimated at about 50 and 150 years, respectively. The effects of this difference cannot be captured over short horizons and require a simulation period of at least 100 years.

Finally, real life practice in the infrastructure/concrete industry is characterized by inertia, whereby a shift from long-established practices, such as the predominant use of OPC for building infrastructure, cannot realistically be achieved over a matter of years and rather would take decades. To reflect the dynamics of such long-term change processes, long simulation periods are needed. With the long-term simulation horizon thus becoming necessary, the problem of uncertain future population and economic growth projections is taken care of by studying several possible scenarios, which is the approach adopted in climate change science as well. Hence, this approach has been used in this study. Accordingly, different assumptions of population growth and per-capita consumption of cement are used to generate four main policy testing scenarios. The model developed gives the user the flexibility to input any other scenario besides those built-in scenarios.

For each of the simulated scenarios, results show that significant reductions in CO_2_ emissions are possible by replacing OPC with UHPC. As discussed in Section 3.2.1, the reduction in the emissions does not occur as a linear proportion of OPC replacement. The reduction—both in annual emissions as well as cumulative emissions—also accounts for the decreased infrastructure demand owing to the use of UHPC having longer service life. Moreover, the reduction achieved is not static for a given policy scenario; rather it evolves over time due to the interaction between obsolete infrastructure getting decommissioned, proportions of OPC and UHPC used for infrastructure with the passage of time, and their respective service lives. Such non-linear and evolving behavior justifies the use of the System Dynamics methodology in this study.

The results also show that not only do the relative fractions of OPC and UHPC used for building infrastructure have a bearing on CO_2_ emissions, but also the initiation and implementation duration of policies have significant implications for these emissions. This would be intuitive for the reader; however, an important contribution of this study is the development of a simulation tool with which the user can easily explore the implications of these policy decisions. Thus, various ‘what if’ scenarios can easily be explored with the developed model, and hence, can aid in policy testing and selection of policies that are deemed crucial and/or implementable.

The System Dynamics model developed herein is flexible and can accommodate further improvements. These can be easily implemented in future research through incremental changes instead of requiring complete redevelopment. For example, the unit intensity of CO_2_ emissions has been assumed as 0.82 kg/kg of cement produced. Although this number can be changed to any other desired value by a model user, once a value is selected, it remains constant throughout the simulation period. Because of continuing improvements in energy efficiency and increasing contribution of cleaner sources of energy (such as wind/solar/waste derived) in national economies, the unit intensity of CO_2_ emissions due to cement manufacture is likely to decrease over the coming years. Similarly, the CO_2_ burden associated with the clinker formation process in the cement manufacture can be reduced through the adoption of supplementary cementitious materials and emerging new technologies. Future work can be aimed at forecasting possible trajectories of CO_2_ intensity of cement production and instead of adopting a single value throughout the simulation, an evolving trend of CO_2_ intensity can be incorporated in the model.

Another simplification adopted in the current model consists of exploring different policy scenarios through exogenous ‘prescribed’ parameters, such as the targeted fraction of UHPC and implementation period to achieve the target values. In a more realistic model, the dynamics of future scenarios can be endogenized. For example, it is reasonable to assume that political/social pressure for the concrete industry to become more sustainable will grow faster if the industry’s track record shows that its carbon emissions are growing unabated. On the other hand, if the industry can demonstrate that its emissions intensity is becoming flatter or even better, decreasing per unit of its economic output, then societal pressure would be relieved. Future work can help improving the model in this respect, i.e., instead of simulating scenarios with fixed policy targets and fixed implementation times, both the UHPC target share and implementation period can be modelled as evolving based on the recent (5–10-year) trends in the carbon emissions of the industry.

In this study, the CO_2_ burden of concretes has been assumed to be due to their cement contents alone. Differences arising due to other variations in their mixture composition have not been taken into account. Disregarding the energy embedded in the constituents of UHPC, such as steel fibers and admixtures, and steam curing methods required for some kinds of UHPC, may result in underestimation of its CO_2_ burden. On the other hand, the reduction in sizes of structural members that is made possible with the use of UHPC will possibly reduce CO_2_ emissions on account of mining, processing, and hauling of materials. Future studies can account for such details and come up with better estimates of CO_2_ emissions associated with the use of UHPC.

Finally, this study has focused on the primary sustainability related aspect of concrete, that is, its CO_2_ emissions. Although this aspect is of significant importance on its own, the concrete industry has to address several other ecological problems. This includes continuing colossal use of natural resources that are growing scarce such as freshwater and sand, and mining virgin resources for extraction of fine and coarse aggregates and raw materials for cement, as well as managing construction and demolition waste. System dynamics is a useful methodology that can be effectively used to further study these aspects of the concrete industry.

## 5. Conclusions

A system dynamics simulation tool was developed in the present study to explore possible reductions in carbon emissions of the concrete industry by shifting from the current predominant use of ordinary Portland cement concrete to ultrahigh performance concrete to satisfy the growing societal need for infrastructure due to population growth, economic development, and the need for replacing the aging existing civil infrastructure. Several scenarios have been tested using this model, which are not intended to predict future trends, but rather to test various plausible policy scenarios. The modular and flexible architecture of the model allows the user to modify various inputs and create new scenarios that can be effectively tested by the model. Based on the simulation results of the model, the following conclusions can be drawn:

1. Although ultrahigh performance concrete (UHPC) is more cement intensive than ordinary concrete (OPC), its much higher mechanical strength allows for using smaller structural members to carry the same load, making it a sustainable contender to normal concrete.
2. The much longer service life of UHPC reduces the need for long-term repair and rehabilitation and the decommissioning and reconstruction of aging structures, which reaps further sustainability benefits in long-term simulation.
3. The effectiveness of sustainability policies depends on how early they are implemented and how long it takes for full policy implementation. Thus, CO_2_ emission savings from UHPC depend of its level of replacing OPC, when this replacement starts, and how long it takes to replace substantial proportions of OPC.
4. Future global CO_2_ emissions from cement production will greatly depend on population growth trends and the per-capita demand for new infrastructure, which is in turn related to economic development. However, new technologies and policies will likely have a significant impact on the end result of the policy simulation.
5. Under certain policy scenarios (L0.5-IV scenario), the replacement of OPC by UHPC can lead to up to 55% savings in annual CO_2_ emissions in the long run. While full replacement of OPC by UHPC is not realistic, the results provide incentive to producing more resilient and sustainable infrastructure to achieve sustainable development goals.
6. The model results show that there is significant potential for CO_2_ reductions, which depend on the various future policy scenarios.
7. More importantly, it is demonstrated that the feedback effects in the system have noticeable impacts on the CO_2_ emission reductions realized. Thus, system dynamics, which was developed specifically to model systems having feedback loops, is shown to be an appropriate methodology for investigating this problem.
8. The developed system dynamics policy testing tool allows the user to explore the outcomes of diverse policy scenarios. It is also modular and flexible, permitting the user to easily improve the model depending of future developments and new data that become available.

## Figures and Tables

**Table 1:** Main scenarios explored in the simulations.

Scenario	Population Growth	Growth Rate for Per Capita Cement Demand
H-0.5	UN high fertility forecast	0.5% per year
H-1.0	UN high fertility forecast	1.0% per year
L-0.5	UN low fertility forecast	0.5% per year
L-1.0	UN low fertility forecast	1.0% per year

**Table 2:** Summary of final results for annual emissions under the four simulation scenarios.

Scenario	Annual CO_2_ Emissions [Billion Metric Tons]				% Reduction in Value Compared to Sub-Scenario I		
	I	II	III	IV	II	III	IV
H-0.5	11.08	9.50	8.63	8.05	14.23	22.07	27.38
H-1.0	18.18	15.84	14.43	13.66	12.85	20.62	24.86
L-0.5	3.79	3.00	2.69	2.30	20.72	36.46	55.18
L-1.0	6.22	5.10	4.60	4.10	17.92	25.95	34.11

Note that initial annual emissions for all the 16 scenarios are 3.03 billion metric tons.

**Table 3:** Summary of results for cumulative emissions under the four simulation scenarios.

Scenario	Cumulative CO_2_ Emissions [Billion Metric Tons]				% Reduction in Value Compared to Sub-Scenario I		
	I	II	III	IV	II	III	IV
H-0.5	651.27	592.17	575.89	535.65	9.08	11.57	17.75
H-1.0	886.19	806.08	778.91	729.07	9.04	12.10	17.73
L-0.5	401.05	364.31	359.76	329.63	9.16	10.30	17.81
L-1.0	522.92	474.76	465.80	429.04	9.21	10.92	17.95

## Data Availability

Data is contained within the article and supplementary material.

## Supplementary Materials

The following are available online at https://www.mdpi.com/1996-1944/14/4/995/s1, the code of the model is provided as a supplementary material along with the final published article. All references to the “user” in this paper refer to anyone who would use the code to run the model in Stella or any other (after necessary modifications) System Dynamics software.

## Abbreviations

CLD	Causal Loop Diagram
CSI	Cement Sustainability Initiative
GHGs	Greenhouse Gases
Gt	Gigaton
IEA	International Energy Agency
IPCC	Intergovernmental Panel on Climate Change
OPC	Ordinary portland cement concrete
SCMs	Supplementary Cementitious Materials
SD	System Dynamics
UHPC	Ultrahigh Performance Concrete
UN	United Nations
WBCSD	World Business Council for Sustainable Development
