# Supplementary material for: Estimating CO2 Emission Savings from Ultrahigh Performance Concrete: A System Dynamics Approach
Source: Materials (Basel). 2021 Feb 20;14(4):995. doi: 10.3390/ma14040995 (PMC7924067; doi:10.3390/ma14040995)
Supplement: Supplementary file 1 [file materials-14-00995-s001.pdf]

C02 Emission sector

CO2_Emitted(t) = CO2_Emitted(t - dt) + (Annual_CO2_emissions) * dt

INIT CO2_Emitted = 0

INFLOWS:

Annual_CO2_emissions = (0.135*OPC_Construction+0.227*UHPC_Construction)*Cement_CO2_production_fraction

Cement_CO2_production_fraction = 0.85

Cement demand sector

Apply_User_Defined_Demand = 0

Cement_Demand = IF Apply_User_Defined_Demand=1 THEN User_Defined_Scenario ELSE IF Scenario=1 THEN Cement_Demand_Forecasts[High05] ELSE IF Scenario=2 THEN Cement_Demand_Forecasts[High1] ELSE IF Scenario=3 THEN Cement_Demand_Forecasts[Low05] ELSE Cement_Demand_Forecasts[Low1]

Cement_Used_for_Concrete_Production = 0.9

OPC_Share = 100-UHPC_Share

Policy_Achieve_Time = 10

Policy_Start_Time = 0

Scenario = 1

Structures_Required = Cement_Used_for_Concrete_Production*(Cement_Demand/.135)-Eq_OPC_Decommis*DT

UHPC_Share = SMTHN(STEP(UHPC_Target_Share,Policy_Start_Time),Policy_Achieve_Time,5,0)

UHPC_Target_Share = 0

Cement_Demand_Forecasts[High05] = GRAPH(TIME)
(0.00, 4100), (5.00, 4452), (10.0, 4829), (15.0, 5226), (20.0, 5627), (25.0, 6038), (30.0, 6468), (35.0, 6922), (40.0, 7403), (45.0, 7908), (50.0, 8434), (55.0, 8984), (60.0, 9562), (65.0, 10175), (70.0, 10825), (75.0, 11512), (80.0, 12229), (85.0, 12803), (90.0, 13524), (95.0, 14273), (100, 15051)

Cement_Demand_Forecasts[High1] = GRAPH(TIME)
(0.00, 4100), (5.00, 4564), (10.0, 5074), (15.0, 5630), (20.0, 6214), (25.0, 6836), (30.0, 7506), (35.0, 8236), (40.0, 9029), (45.0, 9887), (50.0, 10810), (55.0, 11804), (60.0, 12879), (65.0, 14049), (70.0, 15322), (75.0, 16702), (80.0, 18190), (85.0, 19522), (90.0, 21139), (95.0, 22870), (100, 24723)

Cement_Demand_Forecasts[Low05] = GRAPH(TIME)
(0.00, 4100), (5.00, 4376), (10.0, 4624), (15.0, 4850), (20.0, 5066), (25.0, 5265), (30.0, 5441), (35.0, 5588), (40.0, 5704), (45.0, 5792), (50.0, 5856), (55.0, 5896), (60.0, 5911), (65.0, 5902), (70.0, 5869), (75.0, 5815), (80.0, 5740), (85.0, 5615), (90.0, 5476), (95.0, 5313), (100, 5124)

Cement_Demand_Forecasts[Low1] = GRAPH(TIME)
(0.00, 4100), (5.00, 4486), (10.0, 4859), (15.0, 5225), (20.0, 5594), (25.0, 5961), (30.0, 6315), (35.0, 6648), (40.0, 6956), (45.0, 7242), (50.0, 7505), (55.0, 7746), (60.0, 7961), (65.0, 8148), (70.0, 8307), (75.0, 8437), (80.0, 8537), (85.0, 8562), (90.0, 8560), (95.0, 8513), (100, 8417)

User_Defined_Scenario = GRAPH(Time)
(2020, 0.00), (2030, 0.00), (2040, 0.00), (2050, 0.00), (2060, 0.00), (2070, 0.00), (2080, 0.00), (2090, 0.00), (2100, 0.00), (2110, 0.00), (2120, 0.00)

OPC sector

OPC_Infrastructure(t) = OPC_Infrastructure(t - dt) + (OPC_Construction - OPC_Decomissioning) * dt
INIT OPC_Infrastructure = 0
TRANSIT TIME = varies
INFLOW LIMIT = INF
CAPACITY = INF

INFLOWS:
OPC_Construction = (OPC_Share/100)*Structures_Required
OUTFLOWS:
OPC_Decomissioning = CONVEYOR OUTFLOW
TRANSIT TIME = Service_life_of_OPC

Service_life_of_OPC = 50

UHPC Sector

Eq_OPC(t) = Eq_OPC(t - dt) + (Eq_OPC_Constr - Eq_OPC_Decommis) * dt
INIT Eq_OPC = 0
TRANSIT TIME = varies
INFLOW LIMIT = INF
CAPACITY = INF

INFLOWS:
Eq_OPC_Constr = UHPC_Construction*2
OUTFLOWS:
Eq_OPC_Decommis = CONVEYOR OUTFLOW
TRANSIT TIME = Service_life_of_OPC

UHPC_Infrastructure(t) = UHPC_Infrastructure(t - dt) + (UHPC_Construction - UHPC_Decommissioning) * dt
INIT UHPC_Infrastructure = 0
TRANSIT TIME = varies
INFLOW LIMIT = INF
CAPACITY = INF

INFLOWS:
UHPC_Construction = (UHPC_Share/100)*Structures_Required*0.5
OUTFLOWS:
UHPC_Decommissioning = CONVEYOR OUTFLOW
TRANSIT TIME = Service_life__of_UHPC

Service_life__of_UHPC = 150

Not in a sector
